# A Study in Poor-Law Expenditure

**Published:** 1906-04-21

**Authors:** 


### HOSPITAL ADMINISTRATION.

### CONSTRUCTION AND ECONOMICS.

### A STUDY IN POOR-LAW EXPENDITURE.

The announcement that the new President of the Local Government Board has requested the public bodies throughout England to prepare an account of their expenditure will be welcomed by all who think that public expenditure has during late years been going on at a reckless rate. The people grumble, but they pay, and do not inquire too closely where their money goes, and the different methods of book-keeping adopted by different localities make it difficult for an independent investigator to obtain precise information and compare one district with another. If, when he has all the information for which he asks before him, Mr. Burns realises the difficulties created in the way of reform by this diversity, and insists on a standardisation of public book-keeping in a fashion comparable to the "Uniform System of Accounts" now adopted by so many hospitals, he will do a good deed both to officials and ratepayers, as well as to his own successors in office.

In attempting to examine and compare the expenditure of the different Poor-law Unions in the metropolis, the present writer has been hampered at every turn by the different points of view from which the reports of different unions are prepared. One or two refused to give any information, but this was in cases where the union did not publish an annual report, and one could quite understand that the clerical staff could not be asked to plunge into a mass of statistics for the gratification of an outsider. The question in such a case is not, Why was the information refused? but Why is there not a report drawn up at regular intervals, and circulated at least among the Guardians of the Union, and submitted to the local newspapers? It is not to be supposed that if the facts are not gathered together and put before them clearly and concisely, even Guardians—with the possible exception of the Finance Committee—will realise how much they are spending, nor investigate whether the expenditure is wise and necessary; while as for the general body of ratepayers they cannot be otherwise than profoundly ignorant. But even where there is a report, it is often so meagre as to give but little information. And where details are given the variety of opinion as to what is worth telling interferes greatly with the possibility of making a sound generalisation from the figures given. One Union tells the cost of the tea consumed in the workhouse during a year, and another tells the number of the inmates, while only a third sees the necessary correlation between the two sets of facts and therefore gives them both. Thus the following studies are necessarily incomplete, but we believe that, so far as they go they are accurate. They are at least founded on the official statistics.

One of the most popular cries of the moment is that for the equalisation of the poor-rate throughout London. It may be safely said that few of those who raise the cry are aware of the extent to which that rate is at present equalised. If every parish had to bear the burden of its own poor unaided, the poorer districts would undoubtedly be subjected to great hardship, but, as a matter of fact, many of the burdens of the maintenance of the poor are borne in common, each parish contributing according to its rateable value. Each Union contributes to the Metropolitan Common Poor Fund a sum which varies from year to year according to the demands made by the precept of the Local Government Board, but which may be taken at something under 6d. in the £ of the rateable value of the Union. Against the contribution thus levied are set certain charges which are repayable from the Fund. These charges we shall presently detail. But when the amount of these charges exceeds the contribution demanded, not only does the parish not pay over anything, but receives a sum from the Common Poor Fund to make up the necessary amount for these charges. In this way the richer Unions contribute to the necessities of the poorer ones. Thus at Lady Day 1904—the latest statistics we have been able to obtain—the following Unions and parishes received more than they contributed to the extent stated, the contribution being assessed at the rate of fourpence and 153/250ths of a penny in the £ of the annual rateable value of each:—Bermondsey, £19,301; Bethnal Green, £18,405; Camberwell, St. Giles, £11,437; Chelsea, St. Luke, £1,807; Fulham Parish, £1,430; St. George's-in-the East, £10,702; Greenwich, £9,244; Hackney, £9,137; Holborn, £7,417; Islington, St. Mary, £1,503; Lambeth, £1,778; Mile End Old Town, £9,743; St. Pancras, £10,043; Poplar, £20,382; Shoreditch, £8,254; Southwark, £17,374; Stepney, £10,363; Whitechapel, £9,785; Woolwich, £1,455.

Thus the richer parishes of London help the poorer to the extent of £179,362.

The contributing parishes are assessed beyond their own needs as follows:—St. George's, £28,935; St. Giles-in-the-Fields and St. George, Bloomsbury, £12; Hammersmith, £4,388; Hampstead, St. John, £12,532; Kensington, St. Mary Abbott's, £14,253; Lewisham, £3,626; City of London, £76,107; St. Marylebone, £1,045; Paddington, £10,611; Strand, £7,010; Wandsworth, £8,127; Westminster, £11,845; Lincoln's Inn, £648. Lincoln's Inn has no paupers, and its entire assessment is handed over to the Metropolitan Common Poor Fund.

It will thus be seen that London is, in a Poor-law sense, a real entity, and not a mere congeries of independent parishes. It is perhaps desirable that its boundaries should be re-arranged, in view of the development of working-class suburbs just outside the radius, whose inhabitants work within its limits though their homes are outside.

The purposes to which the Metropolitan Common Poor Fund is devoted are the following: —

1. Maintenance of lunatics and insane poor.
2. Maintenance of persons suffering from fever or small-pox.
3. Medicine and medical and surgical appliances.
4. Salaries of officers.
5. Rations of officers.
6. Fees for the registration of births and deaths.
7. Vaccination fees and other expenses of vaccination.
8. Maintenance of children in district and certified schools and training ship, and children boarded out.
9. Relief of vagrants and cost of maintaining vagrant wards.
10. Maintenance of indoor poor (to the extent of 5d. per day for each adult).
11. School fees for children of persons receiving out-door relief.
12. Expenses of the M.A.B. ambulance stations.

Since the advent of free education, the eleventh of these objects has been practically non-existent, but it would be available for fees for education in special schools if necessary.

It will be seen from the list above that little is left on which to spend Poor-law money. But the one thing which remains is at the root of all the agitation. Nothing is drawn from the Metropolitan Common Poor Fund for out-door relief. Therefore those parishes which favour a lavish giving of out-door relief cry out for a unified rate for the whole of London. There are some impulsive Guardians who declare that they will never be satisfied until the workhouse is done away with altogether, and all assistance given in the form of out-door relief. Naturally these amiable persons dislike the thought that such relief must be paid for entirely out of the local rate, and they do not deny that they would give it more frequently and more lavishly if they had all London to draw contributions from. If Poplar, for example, could put its hand into the pocket of the City for all its out-door poor, the latter would certainly receive more per head than they do now. And it is a practical certainty that they would increase in number, for very few of the poor have the least objection to out-door relief, which they can spend as they choose, and for which they have to perform no duties and submit to no discipline.

